# Alcohol Exposure Alters the Ghrelin System: In Vitro Mechanistic Insights Into Impaired Glucose Sensing and Enhanced Ghrelin Secretion

**DOI:** 10.1111/acer.70392

**Published:** 2026-08-04

**Authors:** Sundararajan Mahalingam, Ramesh Bellamkonda, Kusum K. Kharbanda, Ojeshvi Ethiraj, Carol A. Casey, Karuna Rasineni

**Affiliations:** ^1^ Research Service Veterans Affairs Nebraska‐Western Iowa Health Care System Omaha Nebraska USA; ^2^ Department of Biochemistry and Molecular Biology University of Nebraska Medical Center Omaha Nebraska USA; ^3^ Department of Internal Medicine University of Nebraska Medical Center Omaha Nebraska USA

**Keywords:** alcohol use disorder (AUD), alcohol‐associated liver disease (ALD), chronic alcohol, ghrelin, Ghrelinoma cells

## Abstract

**Background:**

Ghrelin, a stomach‐derived orexigenic peptide, typically rises during fasting to stimulate food intake. Chronic alcohol consumption elevates circulating ghrelin, which induces alcohol craving and increases intake in clinical and preclinical models. By increasing alcohol drive, ghrelin contributes to alcohol use disorder (AUD) and accelerates alcohol‐associated liver disease (ALD) by inhibiting insulin secretion and promoting adipose lipolysis. However, the cellular mechanisms by which ethanol dysregulates ghrelin secretion remain unclear. This study investigated how ethanol modulates ghrelin production and disrupts nutrient sensing in ghrelin‐secreting cells.

**Methods:**

Stomach (SG‐1) and pancreatic (PG‐1) ghrelinoma cells were treated with 25 mM or 50 mM ethanol for 48 h, after which ghrelin synthesis and secretion were measured. Further, the interaction between ethanol and other physiological regulators (glucose [10 mM], insulin [20 mM], and palmitic acid [400 μM]) of ghrelin secretion was evaluated. Intracellular glycolytic rate was characterized using Seahorse‐XFe analysis.

**Results:**

Chronic ethanol exposure (48 h) significantly increased ghrelin secretion and the mRNA encoding for ghrelin and ghrelin O‐acyltransferase (GOAT), an enzyme that activates ghrelin. Under normal conditions, high glucose effectively suppressed ghrelin secretion; however, ethanol pretreatment blunted this inhibitory effect. While palmitic acid alone had no effect, its combination with ethanol synergistically enhanced ghrelin secretion. Mechanistically, ethanol‐pretreated cells exhibited a metabolic impairment characterized by increased glucose uptake but significantly reduced basal glycolysis, proton efflux rate (PER), and compensatory glycolytic capacity. This metabolic failure was linked to the profound downregulation of the rate‐limiting enzymes glucokinase, hexokinase, and pyruvate kinase.

**Conclusions:**

Ethanol drives increased ghrelin by disrupting the glucose‐sensing machinery within ghrelin‐secreting cells. By suppressing key glycolytic enzymes, ethanol uncouples the cell from glucose, mimicking a state of starvation despite nutrient availability. Targeting these metabolic pathways may provide a novel therapeutic strategy for the interconnected pathologies of AUD and ALD.

## Graphical abstract

Chronic ethanol exposure increases ghrelin production by impairing glucose sensing in ghrelin‐secreting cells. Ethanol elevates ghrelin and ghrelin O‐acyltransferase expression, blunts glucose‐mediated suppression, and synergizes with fatty acids to enhance secretion. Mechanistically, ethanol suppresses key glycolytic enzymes, reducing glycolytic flux despite increased glucose uptake, thereby mimicking cellular starvation. This metabolic uncoupling links alcohol use disorder to alcohol‐associated liver disease and highlights ghrelin‐cell metabolism as a therapeutic target.

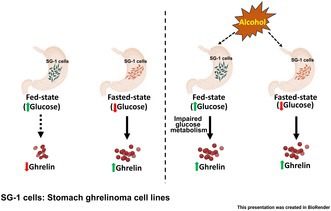

**Abbreviations**
ADH: Alcohol dehydrogenase
ALD: Alcohol‐associated fatty liver disease
ALDH: Aldehyde dehydrogenase
AUD: Alcohol Use Disorder
CYP2E1: Cytochrome P450 2E1
GCK: Glucokinase
GHRL: Ghrelin gene/peptide
GHSR: Growth Hormone Secretagogue Receptor
GOAT: Ghrelin‐O‐Acyltransferase
HK: Hexokinase
PER: Proton Efflux Rate
PG‐1 cells: Pancreatic Ghrelinoma‐1 cells
PK: Pyruvate kinase
SG‐1 cells: Stomach Ghrelinoma‐1 cells
XFe: Extracellular Flux

## 1 Introduction

Excessive alcohol consumption is a major global health concern, contributing significant medical, social, and economic burdens. In 2016, the World Health Organization (WHO) reported that 43% of the global population consumed alcohol, resulting in nearly three million deaths and over 130 million disability‐adjusted life years lost (Global report, World Health Organization 2018). Chronic heavy drinking leads to widespread health complications, including neuropsychiatric disorders, cancer, and organ damage, with the brain and liver being particularly vulnerable (Axley et al. 2019). Prolonged alcohol exposure promotes the development of alcohol use disorder (AUD) and causes hepatic steatosis in approximately 90% of heavy drinkers, which can progress to advanced liver disease with continued alcohol intake (Ramkissoon and Shah 2022).

Therapeutic options for alcohol‐associated liver disease (ALD) remain limited; abstinence and liver transplantation are currently the most effective interventions (Kharbanda et al. 2022; Vannier et al. 2022). However, achieving long‐term abstinence is challenging for individuals with AUD, allowing ongoing alcohol consumption to drive liver injury (Haque and Leggio 2024; Vannier et al. 2022). The pathogenesis of ALD and AUD involves complex, multi‐organ dysfunction, including the gut, brain, adipose tissue, and liver, mediated in part by gut‐derived peptide hormones (Arab et al. 2023; Czerwinska et al. 2021; Farokhnia et al. 2019; Leko and Leggio 2024; Yan et al. 2023).

Among these hormones, ghrelin has gained considerable attention due to its roles in metabolic regulation and addictive behaviors. Ghrelin is a 28‐amino‐acid peptide mainly secreted by gastric P/D1 enteroendocrine cells (Kojima et al. 1999). A small amount is also produced by pancreatic ε‐cells, which make up less than 1% of the pancreatic islet population in adult humans (Dominguez Gutierrez et al. 2018). Ghrelin released from ε‐cells is unlikely to contribute to circulating levels but may function as a local modulator of insulin secretion (Adriaenssens et al. 2016; Digruccio et al. 2016; Sakata et al. 2019). Ghrelin is synthesized as preproghrelin, processed to proghrelin, and subsequently acylated by ghrelin O‐acyltransferase (GOAT) to generate the biologically active, acylated form of ghrelin (Zhu et al. 2006). Ghrelin exerts its effects through the growth hormone secretagogue receptor (GHSR), which is expressed in both central and peripheral tissues. Circulating ghrelin levels rise during fasting and decrease after food intake (Mani and Zigman 2017; Muller et al. 2015).

Alcohol exposure alters circulating ghrelin levels; however, the direction and magnitude of this effect appear to depend on the dose, duration, and physiological context (Deschaine et al. 2022; Koopmann et al. 2012; Leggio et al. 2012; Ralevski et al. 2017). Chronic alcohol intake is consistently associated with elevated total ghrelin in preclinical studies, large population cohorts, and patients with alcohol‐associated cirrhosis or dependence (Farokhnia et al. 2021; Godlewski et al. 2019; Goodyear et al. 2010; Kraus et al. 2005; Rasineni, Thomes, et al. 2019; Wittekind et al. 2018). Elevated ghrelin levels have been associated with increased alcohol craving and consumption, and exogenous ghrelin administration is positively correlated with alcohol craving, increased motivation for self‐administration, and heightened neural reactivity to alcohol cues (Jerlhag et al. 2009; Kim et al. 2005; Koopmann et al. 2012; Leggio et al. 2012, 2014). Our previous work shows that alcohol‐induced ghrelin elevation suppresses insulin secretion from pancreatic β‐cells, leading to increased adipose lipolysis, elevated free fatty acids, and subsequent hepatic steatosis. Ghrelin also directly promotes lipid accumulation in hepatocytes by increasing fatty acid uptake and stimulating de novo lipogenesis (Barazzoni et al. 2005; Li et al. 2014; Rasineni, Kubik, et al. 2019). Furthermore, our recent study showed that chronic alcohol‐induced elevation in ghrelin disrupts GLP‐1 signaling by attenuating GLP‐1 receptor (GLP‐1R)‐mediated signal transduction, through receptor desensitization or receptor–receptor interactions. This interaction between ghrelin further contributes to metabolic dysfunction (Mahalingam et al. 2026).

In this study, we utilized ghrelin‐secreting cell models to directly examine how alcohol modulates hormone secretion. Understanding these pathways is essential, given the hormone's dual role in reward pathways and systemic energy balance. A clearer understanding of how ethanol alters ghrelin production and release may reveal new molecular targets and therapeutic strategies for mitigating the intertwined metabolic and neurobehavioral disturbances characteristic of both AUD and ALD.

## 2 Materials and Methods

### 2.1 Antibodies and Reagents

The antibodies used in the present study (ADH1, ALDH2, CYP2E1, GAPDH, ghrelin, and β‐actin) are listed in Table 1. Secondary antibodies, including goat anti‐rabbit IgG‐HRP and goat anti‐mouse IgG‐HRP, are also detailed in Table 1. The ghrelin ELISA kit was purchased from Millipore Sigma (Cat. No. EZRGRA; St. Louis, MO).

**TABLE 1:** List of antibodies used in this study.

S. No.	Name of the antibody	Cat #	Company	City, State
1	ADH1	5295	Cell Signaling Technology	Danvers, MA
2	ALDH2	MA5‐17029	Invitrogen	Waltham, MA
3	CYP2E1	AB1252	Millipore Sigma	St. Louis, MO
4	GAPDH	2118	Cell Signaling Technology	Danvers, MA
5	β‐Actin	4970	Cell Signaling Technology	Danvers, MA
6	ghrelin	ab85104	abcam	Cambridge, MA
7	Goat anti‐rabbit IgG‐HRP	W401B	Promega	Fitchburg, WI
8	Goat anti‐mouse IgG‐HRP	W402B	Promega	Fitchburg, WI

### 2.2 Cell Culture and Ethanol Treatments

Stomach ghrelinoma‐1 (SG‐1) and pancreatic ghrelinoma‐1 (PG‐1) cells were generously provided by Dr. Jeffrey Zigman (UT Southwestern Medical Center, Dallas, TX). These ghrelinoma cell lines were originally isolated from the mouse stomach and pancreas. SG‐1 and PG‐1 cells were cultured and maintained throughout the experiments according to previously published protocols (Zhao et al. 2010). Briefly, cells were grown in DMEM/F‐12 medium (Corning, Manassas, VA) supplemented with 10% fetal bovine serum (FBS), 100 U/mL penicillin, and 100 μg/mL streptomycin and maintained at 37 C under 5% CO_2_ and 5% O_2_.

For ethanol treatment, approximately 0.75 × 10^6^ cells were seeded into each well of a 6‐well plate containing complete growth media. Upon reaching 50%–60% confluency, the cells were treated with either control growth media or growth media containing ethanol (25 mM or 50 mM) for 24 h or 48 h as reported previously (Mcvicker et al. 2012; Rasineni, Thomes, et al. 2019). Fresh media were replenished every 24 h during ethanol treatment, 15 mM HEPES was added to the media, and plates were sealed to minimize ethanol evaporation. Following incubation, both culture media and cell lysates were collected and stored as described (Zhao et al. 2010). Secreted ghrelin levels in the media were quantified using a ghrelin ELISA kit, and concentration values were normalized to DNA isolated from the corresponding cell samples and expressed as ng/mL media/μg DNA.

### 2.3 Western Blot

Cell homogenates were prepared in ice‐cold lysis buffer (50 mM Tris–HCl, 150 mM NaCl, 0.1% SDS, 0.5% sodium deoxycholate, and 1% NP‐40; pH 7.4) supplemented with a protease inhibitor cocktail (Sigma, St. Louis, MO; P2714‐1BTL). Protein markers and equal protein load of the samples were resolved by 12% SDS‐PAGE and transferred onto nitrocellulose membranes. Membranes were probed with appropriate primary antibodies followed by HRP‐conjugated secondary antibodies, and immunoreactive bands were detected using enhanced chemiluminescence. Images were acquired using a Bio‐Rad ChemiDoc MP imaging system (Bio‐Rad Laboratories, Hercules, CA, USA). Band intensities were quantified using Quantity One software (Bio‐Rad Laboratories), and β‐actin served as the internal loading control.

### 2.4 Glucose Treatment in Ethanol‐Pretreated SG‐1 and PG‐1 Cells

The impact of ethanol on glucose‐mediated ghrelin inhibition was evaluated in both SG‐1 and PG‐1 cells. Cells were seeded at a density of 0.75 × 10^6^ cells per well in 6‐well plates. Upon reaching 50%–60% confluency, cells were treated with or without ethanol‐containing (50 mM) growth media for 48 h. Following this pretreatment, cells were washed with low‐glucose DMEM medium and then incubated with or without 10 mM glucose in low‐glucose medium with 1% FBS for 16 h (Sakata et al. 2012). To investigate the interaction with chronic ethanol, ethanol‐pretreated cells were treated with 10 mM glucose in the presence of 50 mM ethanol. After the treatment period, media and lysates were collected for ghrelin quantification.

### 2.5 Measurement of Ghrelin Secretion Following Fatty Acid and Insulin in Ethanol‐Pretreated Cells

The effects of ethanol on palmitic acid and insulin‐mediated ghrelin secretion were evaluated in SG‐1 cells. Upon reaching confluency, cells were treated with 400 μM palmitic acid in 1% fat‐free BSA media for 16 h (Lu et al. 2012; Zhao et al. 2010). To assess ethanol's effect, SG‐1 cells were pretreated with 50 mM ethanol for 48 h, followed by exposure to palmitic acid together with ethanol for an additional 16 h. Palmitic acid stock solutions were prepared in 1% fat‐free BSA‐containing medium and incubated at 37 C for 30 min before use. In parallel experiments, 20 nM insulin was added to DMEM media with 1% FBS, and cells pretreated with or without ethanol were incubated in the presence or absence of ethanol for 16 h (Sakata et al. 2012). Following treatment, both culture media and cell lysates were collected. Secreted ghrelin levels were quantified and expressed as described above.

### 2.6 RNA Extraction and Quantitative PCR

Total RNA was extracted from SG‐1 and PG‐1 cells using the PureLink RNA Mini Kit (Invitrogen, Carlsbad, CA, USA). cDNA synthesis was performed by reverse‐transcribing 1 μg of total RNA using the high‐capacity cDNA reverse transcription kit (Applied Biosystems, Carlsbad, CA, USA). Quantitative PCR (qPCR) was conducted on a 7500 Fast Real‐Time PCR System (Applied Biosystems, Waltham, MA, USA) using either TaqMan Universal Master Mix II (Thermo Fisher Scientific, Waltham, MA, USA) or iTaq Universal SYBR Green Supermix (Bio‐Rad, Hercules, CA, USA), along with gene‐specific primers sourced from Applied Biosystems or Integrated DNA Technologies (Coralville, IA, USA). Relative gene expression was calculated using the ΔΔCt method, with actin serving as the internal control. Primer sequences from Integrated DNA Technologies are listed in Table 2, except Ghrelin O‐acyltransferase (GOAT) was purchased as a FAM‐labeled primer from Thermo Fisher Scientific (Waltham, MA, USA; Cat # Mm01200389_m1).

**TABLE 2:** Primer sets used in RT‐qPCR analysis.

Protein	Gene Symbol	Forward (5′ to 3′)	Reverse (5′ to 3′)	Accession Number
Ghrelin	ghrl	CCAGGGAGTCACACAGTCTC	CCAGCTTTCCTGGGTTCCTAC	AB060078.1
GLUT1	Glut1	CGTCGTTGGCATCCTTATTG	GAGGCCACAAGTCTGCATTG	NM_011400.4
GLUT4	Glut4	CCTTTCTCATTGGCATCATTTC	CACGGCCAAGACATTGTTG	NM_177709.3
Pyruvate kinase1	Pklr	AGTCCCACACTTTGGAAGCA	TGGGAGAAGTTGAGTCGTGC	NM_013631.2
Glucokinase1	Gck1	CCGTGATCCGGGAAGAGAA	GGGAAACCTGACAGGGATGAG	L41631.1
Hexokinase1	Hk1	CGAGCCTAGATTGCGGAATC	AAATTCCTCTCTCCTTTTTACAGCAT	NM_001146100.2

### 2.7 Glucose Uptake Assay

The glucose uptake capacity of SG‐1 cells was assessed using the Glucose Uptake‐Glo Assay Kit (Cat # J1341; Promega, Wisconsin, USA), following the manufacturer's protocol. This assay is based on cellular uptake of 2‐deoxyglucose (2‐DG) via glucose transporters and its subsequent phosphorylation to 2‐DG‐6‐phosphate, which accumulates intracellularly. Following cell lysis, 2‐DG‐6‐phosphate is enzymatically converted to generate NADPH, which drives a luciferase reaction producing luminescence proportional to glucose uptake. Briefly, 0.75 × 10^6^ cells were seeded per well in 6‐well plates for ethanol treatment as described above. When cells reached approximately 50%–60% confluency, they were treated with 50 mM ethanol for 48 h. Following treatment, the culture medium was removed, cells were washed with phosphate‐buffered saline (PBS), harvested, and counted. Cells from untreated control and 50 mM ethanol–treated groups were then seeded at a density of 15,000 cells per well in 96‐well plates and incubated for 3 h at 37 C in a humidified atmosphere containing 5% CO_2_ and 5% O_2_. To initiate the assay, 50 μL of freshly prepared 1 mM 2‐deoxyglucose (2DG) was added to each well, followed by brief shaking and a 10‐min incubation at room temperature. Subsequently, 25 μL of stop buffer was added and shaken briefly, followed by 25 μL of neutralization buffer with another brief shake. Then, 100 μL of 2‐deoxy‐D‐glucose 6‐phosphate detection reagent was added and mixed gently. Plates were incubated at room temperature for 2 h. Luminescence was recorded using a luminometer with an integration time of 0.3–1 s.

### 2.8 Seahorse XFe24 Glycolytic Rate

SG‐1 cells were seeded at a density of 80,000 cells per well in a Seahorse XFe24 24‐well plate and cultured at 37 C in a humidified atmosphere containing 5% CO_2_ and 5% O_2_. Upon reaching 50%–60% confluency, cells were treated with 50 mM ethanol in complete growth media for 48 h. At least 24 h prior to the assay, the Seahorse XFe port injector sensor cartridge was hydrated by placing it in 1 mL of Seahorse XFe calibrant fluid and incubating overnight in a non‐CO_2_ incubator. On the day of the assay, the culture medium was aspirated and replaced with Seahorse XFe DMEM base medium supplemented with 2 mM glutamine, 1 mM pyruvate, and 10 mM glucose (pH 7.4). Plates were then incubated at 37 C in a non‐CO_2_ environment. Port injections were prepared using the same base medium. Port A was loaded with rotenone/antimycin A (Rot/AA) to achieve a final concentration of 0.5 μM post‐injection, while Port B contained 2‐deoxyglucose (2‐DG) at a final concentration of 50 mM. The injection strategy was designed using Wave software and uploaded to the Seahorse XFe system. Following this, the sensor cartridge and cell plate were inserted into the instrument to initiate data acquisition. Data was normalized to DNA in the wells.

### 2.9 Statistical Analysis

Results are expressed as mean values ± SEM. Data were analyzed using one‐way ANOVA, followed by the Tukey test. Comparison between 2 groups was analyzed using the Student's *t*‐test. A *p*‐value of < 0.05 was considered statistically significant.

## 3 Results

### 3.1 Alcohol Treatment Enhances Ghrelin Secretion

**FIGURE 1:**
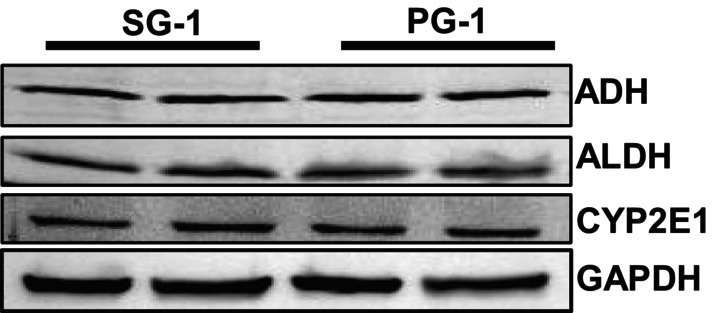
Alcohol metabolizing enzymes in ghrelinoma cells. Representative Western blot images showing alcohol dehydrogenase (ADH), aldehyde dehydrogenase (ALDH), and Cytochrome P450 2E1 (CYP2E1) protein expression in SG‐1 and PG‐1 cells. *n* = 4.

For in vitro studies, we have used ghrelin‐secreting cell lines (ghrelinoma cell lines) derived from the mouse stomach (SG‐1) and pancreas (PG‐1). First, to confirm the presence of alcohol‐metabolizing enzymes, we analyzed the expression of alcohol dehydrogenase (ADH), aldehyde dehydrogenase (ALDH), and Cytochrome P450 2E1 (CYP2E1) in SG‐1 and PG‐1 cells using Western blotting. Western blot analysis confirmed the presence of ADH and ALDH proteins along with CYP2E1 in both cell lines, with similar expression levels observed in SG‐1 and PG‐1 cells (Figure 1).

**FIGURE 2:**
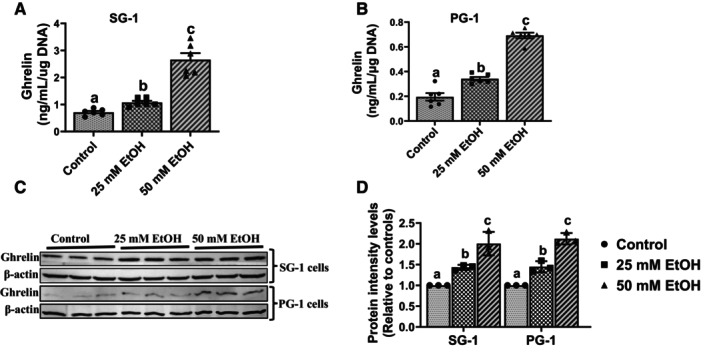
Ethanol treatment elevates ghrelin secretion in SG‐1 and PG‐1 cells. The level of ghrelin secretion in SG‐1 and PG‐1 cells after 25 mM EtOH or 50 mM EtOH for 48 h EtOH exposure for 48 h. (A) Ghrelin levels in the growth media of SG‐1 cells. (B) Ghrelin levels in the growth media of PG‐1 cells. Ghrelin concentration was normalized to DNA isolated from the corresponding cell samples and expressed as ng/mL media/μg DNA. Values are mean ± SEM, (*n* = 6). Values that do not share a common letter are statistically different, *p* ≤ 0.05. (C) Representative western blot images showing ghrelin protein expression in SG‐1 and PG‐1 cells incubated in control medium or treated with 25 mM or 50 mM ethanol (EtOH) for 48 h. (D) Densitometric values of ghrelin protein after normalizing with β‐Actin. Values are mean ± SEM, (*n* = 3). Values that do not share a common letter within the bar graph are statistically different, *p* ≤ 0.05.

**FIGURE 3:**
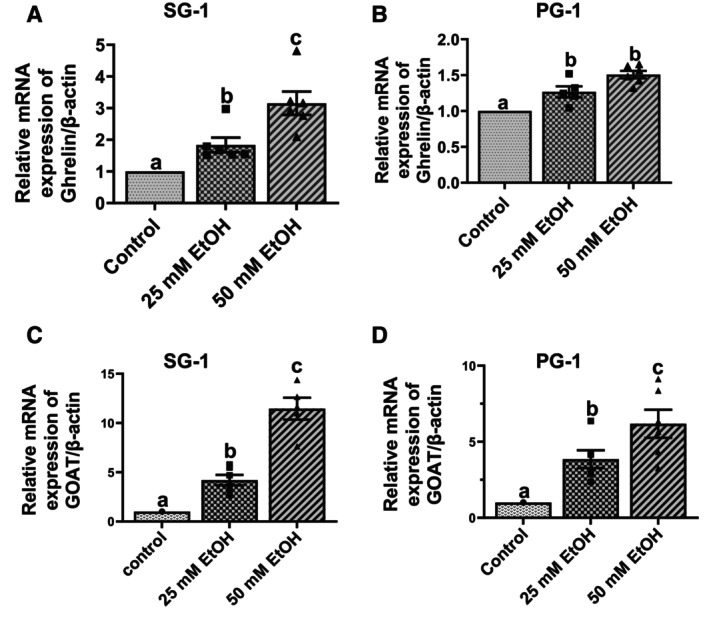
Ethanol treatment increases mRNA expression of the genes encoding ghrelin synthesis and activation in SG‐1 and PG‐1 cells. Expression of mRNA encoding ghrelin in SG‐1 cells (A) and PG‐1 cells (B) treated with 25 mM EtOH or 50 mM EtOH for 48 h. Expression of mRNA encoding ghrelin O‐acyltransferase (GOAT) in SG‐1 cells (C) and PG‐1 cells (D) treated with 25 mM EtOH or 50 mM EtOH for 48 h. Values are mean ± SEM, (*n* = 6). Values that do not share a common letter within the bar graph are statistically different, *p* ≤ 0.05.

To determine whether alcohol influences ghrelin secretion, we measured acylated ghrelin (active ghrelin), hereafter called ghrelin, in culture media collected from SG‐1 and PG‐1 cells after 24 and 48 h of ethanol exposure. After 24 h, ethanol had no significant effect on ghrelin secretion. In contrast, 48‐h ethanol treatment significantly increased ghrelin release. As shown in Figure 2A,B, basal ghrelin secretion from control SG‐1 cells is ~1 ng/mL/μg DNA, while PG‐1 cells secrete ~0.2–0.3 ng/mL/μg DNA under basal conditions. In both cell lines, ethanol treatment induced a significant increase in ghrelin secretion. Cells treated with 50 mM ethanol exhibited the highest ghrelin secretion, showing 3.5‐fold and 3.6‐fold increases compared with controls in SG‐1 and PG‐1 cells, respectively, and greater secretion than the 25 mM ethanol groups. These data indicate that ethanol stimulates ghrelin secretion in both SG‐1 and PG‐1 cells in a concentration‐dependent manner. Consistent with the increase in secretion, cellular ghrelin levels also increased with ethanol treatment and showed a dose‐dependent pattern (Figure 2C,D). Furthermore, to determine whether enhanced ghrelin levels were associated with transcriptional regulation, we measured expression of mRNA encoding ghrelin using qRT‐PCR. Ethanol treatment for 48 h significantly upregulated ghrelin transcripts in both SG‐1 and PG‐1 cells; the increase was dose‐dependent in SG‐1 cells, but not in PG‐1 cells. (Figure 3A,B). Similar to the ghrelin gene, ethanol treatment also significantly increased expression of mRNA encoding GOAT, the enzyme responsible for ghrelin activation, in both SG‐1 and PG‐1 cells in a dose‐dependent manner (Figure 3C,D). Together, these data suggest that ethanol promotes ghrelin synthesis and activation at the transcriptional level.

### 3.2 Ethanol Disrupts Glucose‐Dependent Suppression of Ghrelin Secretion

**FIGURE 4:**
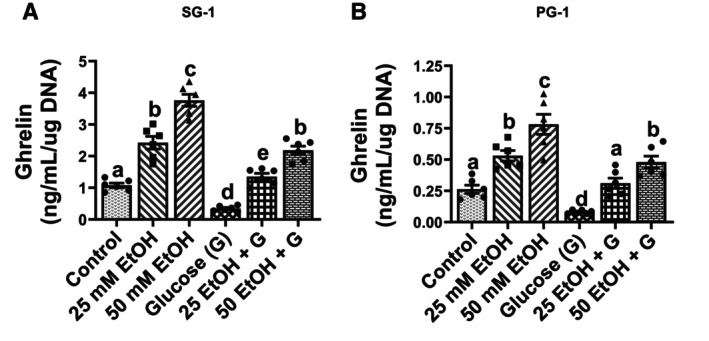
Ethanol disrupts glucose‐dependent suppression of ghrelin secretion. Ghrelin secretion in SG‐1 cells (A) and PG‐1 cells (B) pretreated with control medium or with 25 mM or 50 mM ethanol (EtOH) for 48 h and subsequently treated with 10 mM glucose for 16 h. Ethanol was maintained in the culture medium during the glucose treatment period for ethanol‐pretreated cells. Ghrelin concentration in the media was normalized to DNA isolated from the corresponding cell samples and expressed as ng/mL medium/μg DNA. Values are mean ± SEM (*n* = 6). Values that do not share a common letter within the bar graph are statistically different, *p* ≤ 0.05.

Circulating ghrelin levels typically decline after meals, and previous studies have shown that high glucose suppresses ghrelin secretion in these ghrelinoma cells (Sakata et al. 2012; Steensels et al. 2016). To determine whether ethanol interferes with this glucose‐dependent inhibition, SG‐1 and PG‐1 cells were pre‐exposed to ethanol for 48 h and subsequently treated with 10 mM glucose (which is equivalent to a blood glucose level of 180 mg/dL), with and without ethanol. As expected, glucose alone significantly reduced ghrelin secretion compared with untreated controls (*p* ≤ 0.001) in both SG‐1 and PG‐1 cells. However, in ethanol‐treated cells, glucose was unable to suppress ghrelin secretion to the same extent (Figure 4A,B). Ethanol robustly increased ghrelin secretion, and although glucose significantly reduced this ethanol‐induced increase, ghrelin levels remained higher than in glucose‐treated controls. Collectively, these results demonstrate that ethanol impairs the normal glucose‐dependent suppression of ghrelin secretion in both SG‐1 and PG‐1 cells.

### 3.3 Effects of Insulin and Long‐Chain Fatty Acid on Ethanol‐Induced Ghrelin Secretion in SG‐1 Cells

**FIGURE 5:**
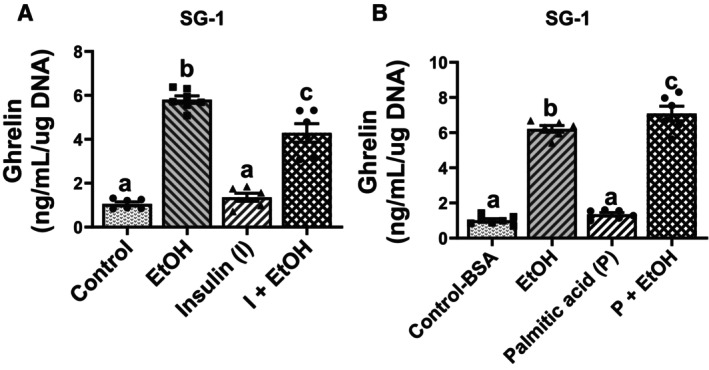
Effects of insulin and palmitic acids on ethanol‐induced ghrelin secretion in SG‐1 cells. SG‐1 cells were pretreated with control or 50 mM ethanol containing media (EtOH) for 48 h and subsequently treated with insulin (A) or BSA‐conjugated palmitic acid (B) for 16 h. Ethanol was maintained in the culture medium during the 20 nM insulin or 400 μM palmitic acid treatment period in ethanol‐pretreated cells. Ghrelin concentration in the media was normalized to DNA isolated from the corresponding cell samples and expressed as ng/mL medium/μg DNA. Values are mean ± SEM (*n* = 6). Values that do not share a common letter within the bar graph are statistically different, *p* ≤ 0.05.

Given the ability of ethanol to disrupt glucose‐mediated inhibition of ghrelin secretion, we next examined whether other metabolic regulators, specifically insulin and long‐chain fatty acids, modify ethanol‐induced ghrelin release (Engelstoft et al. 2013; Lu et al. 2012; Naughton et al. 2018; Zhao et al. 2010). Because SG‐1 and PG‐1 cells showed comparable changes in ghrelin secretion in response to glucose and ethanol, and because the stomach is the major physiological source of circulating ghrelin, all subsequent mechanistic experimental data were presented from stomach‐derived ghrelinoma cells (SG‐1 cells). SG‐1 cells were treated with control or ethanol‐containing media for 48 h and then treated with insulin or palmitic acid in the presence or absence of ethanol for 16 h (only pre‐exposed cells received ethanol during this 16 h treatment). As shown in Figure 5, ethanol treatment alone significantly increased ghrelin secretion compared with control. Insulin alone did not alter ghrelin levels; however, it significantly reduced ethanol‐induced ghrelin secretion (*p* ≤ 0.003) (Figure 5A). As reported previously in ghrelinoma cells (Zhao et al. 2010), palmitic acid alone did not affect ghrelin secretion; however, its combination with ethanol produced a substantial increase (Figure 5B). These findings indicate that ethanol strongly stimulates ghrelin secretion and that this effect is further amplified by long‐chain fatty acids.

### 3.4 Effect of Ethanol on Glucose Uptake in Ghrelinoma Cells

**FIGURE 6:**
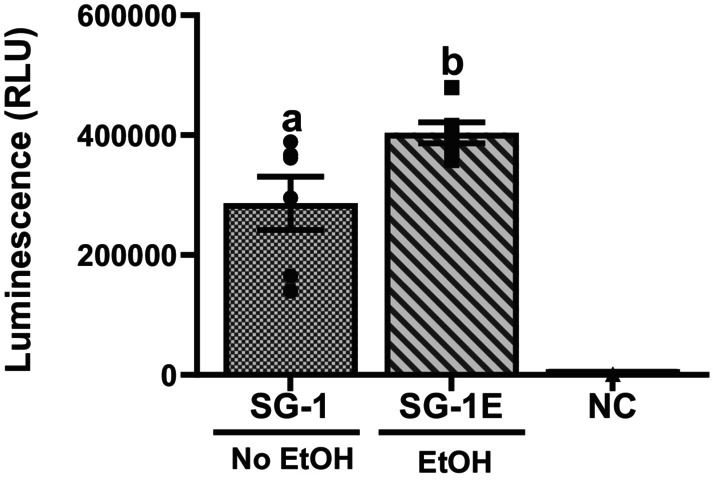
Effect of ethanol treatment on glucose uptake in ghrelinoma cells. Stomach ghrelinoma cells were pretreated with control or ethanol (EtOH) containing media for 48 h. After 48 h of culture, cells were washed with PBS and subjected to a glucose uptake assay. After adding the 2‐deoxyglucose‐6‐phosphate detection reagent, luminescence (RLU) was measured. Negative control (NC) is without substrate 2‐deoxyglucose. Values are mean ± SEM (*n* = 6). Values that do not share a common letter within the bar graph are statistically different, *p* ≤ 0.05.

Glucose is a direct negative regulator of ghrelin secretion, likely in part by signaling cellular energy sufficiency; therefore, any ethanol‐induced change in glucose handling could uncouple the normal postprandial suppression of ghrelin. Because we observed that ethanol blunts glucose‐mediated inhibition of ghrelin secretion, this raised the possibility that ethanol disturbs glucose metabolism within ghrelin‐secreting cells. We therefore tested whether ethanol alters glucose metabolism as a potential mechanism linking chronic ethanol exposure to increased ghrelin release under both fed and fasting conditions. To address this, glucose uptake was assessed in SG‐1 cells treated with control media or ethanol‐containing media for 48 h using a luminescence‐based assay. As shown in Figure 6, ethanol treatment significantly increased glucose uptake compared with controls (*p* < 0.05). No luminescence was detected in the negative controls (NC; without 2‐deoxyglucose treatment). These results indicate that ethanol enhances glucose uptake in ghrelinoma cells. A similar increase in glucose uptake with ethanol treatment was also observed in PG‐1 cells (Figure S1).

**FIGURE 7:**
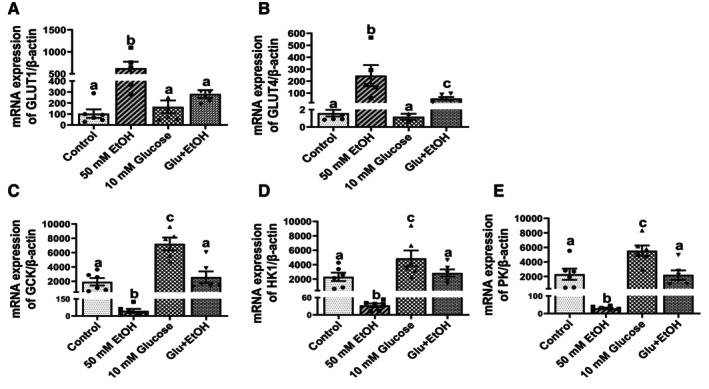
Effect of ethanol treatment on glucose transporters and glycolytic enzymes in ghrelinoma cells. Stomach ghrelinoma cells were pretreated with control or ethanol (EtOH) containing media for 48 h and subsequently treated with 10 mM glucose for 16 h. Expression of mRNA encoding (A) glucose transporter 1 (GLUT1), (B) glucose transporter 4 (GLUT4). (C–E) Expression of mRNA encoding glycolytic enzymes glucokinase (GCK), hexokinase (HK1), and pyruvate kinase (PK). Values are mean ± SEM, (*n* = 6). Values that do not share a common letter within the bar graph are statistically different, *p* ≤ 0.05.

Further, we measured the expression levels of glucose transporters GLUT1 and GLUT4, which are known to be present in ghrelinoma cells (Sakata et al. 2012). In ethanol‐pre‐treated SG‐1 cells, both transporters were increased compared with control cells. In contrast, when cells were treated with 10 mM glucose for 16 h, we did not observe glucose‐induced changes in GLUT1 or GLUT4 expression in control cells (Figure 7A,B). However, ethanol‐pre‐treated cells continued to show elevated transporter expression even after glucose treatment, although the increase was significantly lower than in ethanol‐exposed cells without glucose treatment (Figure 7A,B). Further, we examined the expression of key glycolytic enzymes, glucokinase, hexokinase, and pyruvate kinase (Figure 7C–D). In ethanol‐pretreated cells, the expression of all three enzymes was significantly lower than in control cells. In contrast, glucose treatment induced the expression of these enzymes in control cells; however, in ethanol‐treated cells, glucose increased enzyme expression only back to basal control levels.

### 3.5 Ethanol Treatment Reduces Glycolytic Activity in Stomach Ghrelinoma Cells

**FIGURE 8:**
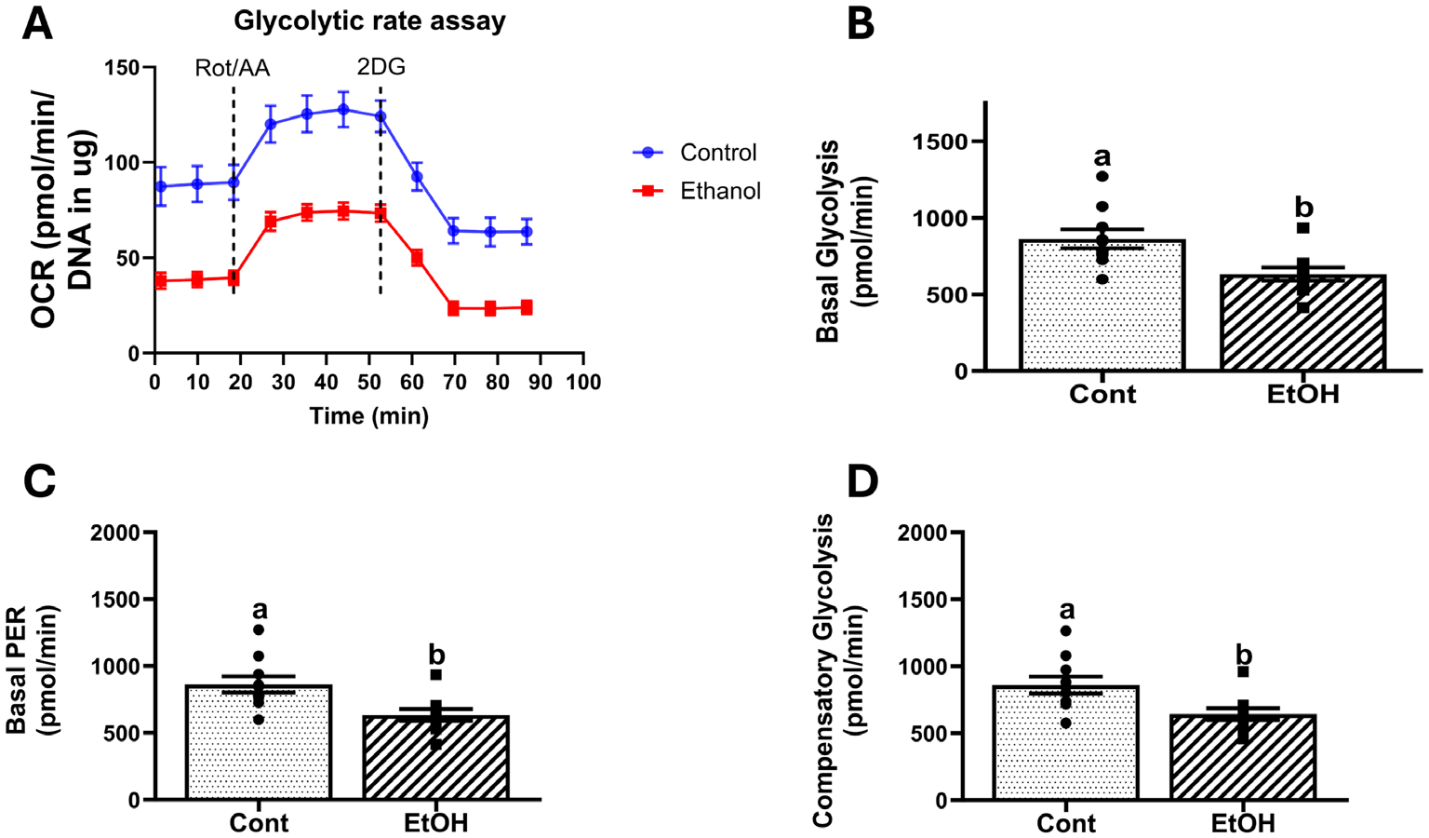
Effect of ethanol treatment on glycolysis in ghrelinoma cells Stomach ghrelinoma cells were pretreated with control or ethanol (EtOH) containing media for 48 h. After 48 h of culture, cells were washed with Seahorse XFe DMEM base medium and subjected to Seahorse glycolytic rate assay using the standard protocol. (A) Overview of Seahorse glycolytic rate assay. (B) Basal glycolysis. (C) Basal proton efflux rate (PER), an indicator of glycolytic flux. (D) Compensatory glycolysis respiration rate. Results are from three independent experiments. Values are mean ± SEM. Values that do not share a common letter within the bar graph are statistically different, *p* ≤ 0.05.

To assess the impact of ethanol exposure on glycolysis, which is utilized by ghrelin‐secreting cells (Sakata et al. 2012), we performed a Seahorse glycolytic assay. Oxygen consumption rate (OCR) was monitored over time following sequential injections of Rotenone/Antimycin A (Rot/AA) and 2‐deoxyglucose (2DG). As shown in Figure 8A, control cells exhibited a marked increase in glycolytic activity after Rot/AA injection, which was significantly attenuated upon 2DG addition. In contrast, ethanol‐treated cells displayed a lower glycolytic response throughout the assay, indicating impaired glycolytic capacity.

Quantitative analysis further demonstrated that basal glycolysis was significantly reduced in ethanol‐treated cells compared to controls (Figure 8B). Similarly, basal proton efflux rate (PER), an indicator of glycolytic flux, was lower in ethanol‐treated cells (Figure 8C). Compensatory glycolysis, measured after mitochondrial inhibition, was also markedly diminished by ethanol treatment (Figure 8D). Collectively, these results indicate that ethanol suppresses both basal and compensatory glycolytic pathways, suggesting a metabolic disconnect in which glucose enters the cell but is inefficiently utilized.

## 4 Discussion

Ghrelin acts as a vital homeostatic link between the body's energy status and behavior. By activating its receptor, GHSR, ghrelin influences food intake, gastrointestinal motility, and energy expenditure, while also participating in systemic energy homeostasis by regulating insulin secretion (Dezaki et al. 2004, 2008). Recent research clearly demonstrates that the ghrelin system is also integrated into the brain's reward and addiction pathways, particularly in the context of AUD (Farokhnia et al. 2018; Leggio et al. 2012, 2014). In both humans and animal models, manipulating the ghrelin axis alters alcohol seeking and cue reactivity, and GHSR antagonism or inverse agonism reduces multiple alcohol‐related behaviors, underscoring the translational potential of this pathway (Farokhnia et al. 2020; Jerlhag et al. 2009; Kaur and Ryabinin 2010; Lee, Farokhnia, et al. 2020; Lee, Tapocik, et al. 2020; Stevenson et al. 2015, 2016). Our previous research identified that the chronic alcohol‐induced rise in circulating ghrelin levels is a primary driver of hepatic pathology, promoting dysfunction across a complex pancreas‐adipose‐liver axis (Rasineni, Thomes, et al. 2019). Specifically, we demonstrated that pharmacological or genetic GHSR knockdown protects against the development of ALD (Mahalingam et al. 2024; Rasineni, Kubik, et al. 2019). These observations underscore a pivotal link between dysregulated ghrelin signaling and the progression of interconnected metabolic and behavioral disorders.

The present study aligns with clinical and preclinical observations showing that chronic alcohol administration increases circulating ghrelin levels (Farokhnia et al. 2021; Godlewski et al. 2019; Goodyear et al. 2010; Kraus et al. 2005; Rasineni, Thomes, et al. 2019; Wittekind et al. 2018). We observed that 48‐h ethanol exposure significantly increased ghrelin secretion at both 25 and 50 mM conditions. This elevation appears to stem from increased synthesis and maturation, evidenced by increased expression of the ghrelin gene and ghrelin‐activating enzyme, GOAT. Notably, we did not observe significant changes following 24 h of exposure, consistent with reports that short‐term (6 h) alcohol exposure fails to alter secretion (Deschaine et al. 2022). This temporal sensitivity mirrors clinical findings: acute ethanol exposure in healthy humans often reduces circulating ghrelin (Calissendorff et al. 2005, 2006; Deschaine et al. 2022; Leggio et al. 2012; Ralevski et al. 2017), whereas elevated hormone levels characterize chronic consumption and alcohol‐associated cirrhosis (Goodyear et al. 2010; Kraus et al. 2005).

A major objective of this study was to determine how ethanol dysregulates ghrelin secretion. Under normal physiology, circulating ghrelin falls during the postprandial period and rises during fasting to signal energy need; however, after chronic ethanol exposure, we observed elevated ghrelin under both fed and fasting conditions. We therefore examined whether ethanol disrupts nutrient‐sensing in ghrelin‐secreting cells and how it interacts with physiological regulators of ghrelin, including glucose, insulin, and long‐chain fatty acids.

As reported previously, elevated glucose (10 mM) significantly decreased ghrelin secretion (Sakata et al. 2012; Steensels et al. 2016), consistent with glucose acting as an energy‐sufficiency signal to ghrelin cells. In our model, insulin and palmitic acid alone (16 h treatment) did not alter secretion, a finding previously reported in these ghrelinoma cells (Zhao et al. 2010). However, other in vivo and in vitro studies have shown that the administration of other long‐chain fatty acids, such as linolenic and palmitoleic acids, or insulin, suppresses circulating ghrelin (Engelstoft et al. 2013; Lu et al. 2012; Naughton et al. 2018; Shankar et al. 2021). This discrepancy may be attributable to differences in treatment duration, the specific lipid species utilized, or the timing of sample collection. Importantly, we found that the presence of ethanol fundamentally shifted this nutrient regulation: glucose‐mediated inhibition was blunted, indicating that ethanol interferes with the cell's nutrient‐sensing machinery and downstream inhibitory signaling. Moreover, although palmitic acid alone was inert, the combination of ethanol and palmitic acid significantly increased ghrelin secretion relative to ethanol alone. This synergistic response parallels hepatocyte studies in which fatty acids are modest in isolation but amplify inflammatory responses when combined with alcohol (Mcvicker et al. 2012). Together, these findings suggest that elevated circulating free fatty acids from adipose lipolysis during chronic alcohol use (Kang et al. 2007; Zhong et al. 2012) may further enhance ghrelin secretion through additive or synergistic mechanisms. Further, given that ghrelin is reduced in metabolic dysfunction‐associated steatotic liver disease (MASLD) but has not been evaluated in metabolic dysfunction and alcohol‐associated liver disease (MetALD), a steatotic liver disease driven by the combined effects of metabolic dysfunction and alcohol consumption, this lipid‐dependent amplification may represent a potentially distinct hormonal signature of alcohol‐driven metabolic injury. However, future studies in both clinical and experimental MetALD models are needed to clarify this relationship.

In this study, we also observed that high glucose suppressed ghrelin secretion in control cells but not in ethanol‐pretreated cells. The observation provides mechanistic insight into how ethanol induces a state of metabolic dysregulation. We demonstrate that chronic ethanol exposure directly enhances ghrelin secretion by disrupting intracellular glucose metabolism, effectively decoupling hormone release from the cell's energy status. Our findings indicate that ethanol impairs glycolytic flux and reduces the expression of key enzymes, glucokinase, hexokinase, and pyruvate kinase. By suppressing these rate‐limiting steps, ethanol mimics a cellular starvation state even when glucose is abundant, particularly in the presence of increased glucose transporter expression and glucose uptake. Seahorse analysis confirmed that ethanol‐pretreated cells exhibited reduced baseline glycolysis and diminished glycolytic capacity. The observed reduction in proton efflux rate (PER) and the attenuated response to mitochondrial inhibition further indicate that ethanol‐treated cells are unable to efficiently utilize glucose for energy. Consequently, the ghrelin cell perceives a low‐energy state, triggering a starvation signal that promotes sustained hormone secretion.

The downregulation of glucokinase is particularly significant, as glucokinase serves as a primary metabolic sensor in various endocrine cells (Gersing et al. 2025; Postic et al. 2001; Sakata et al. 2012). Its suppression by ethanol likely prevents the cell from accurately sensing and responding to glucose concentrations. Notably, while glucose treatment induced glucokinase, hexokinase, and pyruvate kinase expression in control cells, ethanol‐pretreated cells failed to mount a comparable response. The interplay between alcohol‐induced metabolic impairments observed here warrants further investigation to identify the upstream driver(s) of this enzymatic downregulation.

Increased ghrelin resulting from this cellular dysfunction has profound systemic implications. Elevated ghrelin not only heightens alcohol craving but also promotes ALD development. By suppressing insulin secretion and promoting adipose lipolysis, the alcohol‐ghrelin axis increases the flux of free fatty acids to the liver, leading to advanced steatosis.

## 5 Conclusions

In summary, our data indicate that the ethanol‐induced elevation in ghrelin is driven, at least in part, by a failure of glycolytic‐sensing within ghrelin‐secreting cells. By suppressing the expression of key glycolytic enzymes, glucokinase, hexokinase, and pyruvate kinase, ethanol effectively uncouples the cell from the suppressive effects of glucose, leading to sustained hormone secretion. However, these findings are derived from in vitro cell models, and further studies are needed to confirm the physiological relevance of these mechanisms in vivo.

Overall, this work provides mechanistic insight into how ethanol may dysregulate ghrelin biology and highlights potential metabolic pathways that could be explored as therapeutic targets in alcohol use disorder and alcohol‐associated liver disease.

## Author Contributions

S. Mahalingam: Methodology, formal analysis, investigation, drafting manuscript. R. Bellamkonda: Methodology, formal analysis, investigation. K.K. Kharbanda: Conceptualization, manuscript writing and editing. O. Ethiraj: Methodology. C.A. Casey: Editing. K. Rasineni: Conceptualization, formal analysis, funding acquisition, project administration, resources, supervision, visualization, writing – original draft, editing. All authors contributed to the content of the paper and the development of the final draft and approved the final submitted version.

## Funding

This work was supported by the National Institutes of Health/NIAAA funding R01AA028504 (KR), P50AA030407–5131 (KKK and KR), R01AA020735 (CAC) and Merit Review grant I01BX006064 (KKK) and support from the US Department of Veterans Affairs, Biomedical Laboratory Research, and Development Service (CAC and KKK).

## Conflicts of Interest

The authors declare no conflicts of interest.

## Supporting information


**Figure S1:** Effect of ethanol treatment on glucose uptake in ghrelinoma cells. Pancreatic ghrelinoma.

## Data Availability

Research data are not shared.

## References

[acer70392-bib-0001] Adriaenssens, A. E. , B. Svendsen , B. Y. Lam , et al. 2016. “Transcriptomic Profiling of Pancreatic Alpha, Beta and Delta Cell Populations Identifies Delta Cells as a Principal Target for Ghrelin in Mouse Islets.” Diabetologia 59: 2156–2165. 27390011 10.1007/s00125-016-4033-1 PMC5016554

[acer70392-bib-0002] Arab, J. P. , G. Addolorato , P. Mathurin , and M. R. Thursz . 2023. “Alcohol‐Associated Liver Disease: Integrated Management With Alcohol Use Disorder.” Clinical Gastroenterology and Hepatology 21: 2124–2134. 36858144 10.1016/j.cgh.2023.02.017

[acer70392-bib-0003] Axley, P. D. , C. T. Richardson , and A. K. Singal . 2019. “Epidemiology of Alcohol Consumption and Societal Burden of Alcoholism and Alcoholic Liver Disease.” Clinics in Liver Disease 23: 39–50. 30454831 10.1016/j.cld.2018.09.011

[acer70392-bib-0004] Barazzoni, R. , A. Bosutti , M. Stebel , et al. 2005. “Ghrelin Regulates Mitochondrial‐Lipid Metabolism Gene Expression and Tissue Fat Distribution in Liver and Skeletal Muscle.” American Journal of Physiology. Endocrinology and Metabolism 288: E228–E235. 15328073 10.1152/ajpendo.00115.2004

[acer70392-bib-0005] Calissendorff, J. , O. Danielsson , K. Brismar , and S. Rojdmark . 2005. “Inhibitory Effect of Alcohol on Ghrelin Secretion in Normal Man.” European Journal of Endocrinology 152: 743–747. 15879360 10.1530/eje.1.01905

[acer70392-bib-0006] Calissendorff, J. , O. Danielsson , K. Brismar , and S. Rojdmark . 2006. “Alcohol Ingestion Does Not Affect Serum Levels of Peptide YY but Decreases Both Total and Octanoylated Ghrelin Levels in Healthy Subjects.” Metabolism 55: 1625–1629. 17142135 10.1016/j.metabol.2006.08.003

[acer70392-bib-0007] Czerwinska, M. , K. Czarzasta , and A. CUDNOCH‐Jedrzejewska . 2021. “New Peptides as Potential Players in the Crosstalk Between the Brain and Obesity, Metabolic and Cardiovascular Diseases.” Frontiers in Physiology 12: 692642. 34497533 10.3389/fphys.2021.692642 PMC8419452

[acer70392-bib-0008] Deschaine, S. L. , M. Farokhnia , A. GREGORY‐Flores , et al. 2022. “A Closer Look at Alcohol‐Induced Changes in the Ghrelin System: Novel Insights From Preclinical and Clinical Data.” Addiction Biology 27: e13033. 33908131 10.1111/adb.13033 PMC8548413

[acer70392-bib-0009] Dezaki, K. , H. Hosoda , M. Kakei , et al. 2004. “Endogenous Ghrelin in Pancreatic Islets Restricts Insulin Release by Attenuating Ca2+ Signaling in Beta‐Cells: Implication in the Glycemic Control in Rodents.” Diabetes 53: 3142–3151. 15561944 10.2337/diabetes.53.12.3142

[acer70392-bib-0010] Dezaki, K. , H. Sone , and T. Yada . 2008. “Ghrelin Is a Physiological Regulator of Insulin Release in Pancreatic Islets and Glucose Homeostasis.” Pharmacology & Therapeutics 118: 239–249. 18433874 10.1016/j.pharmthera.2008.02.008

[acer70392-bib-0011] Digruccio, M. R. , A. M. Mawla , C. J. Donaldson , et al. 2016. “Comprehensive Alpha, Beta and Delta Cell Transcriptomes Reveal That Ghrelin Selectively Activates Delta Cells and Promotes Somatostatin Release From Pancreatic Islets.” Molecular Metabolism 5: 449–458. 27408771 10.1016/j.molmet.2016.04.007 PMC4921781

[acer70392-bib-0012] Dominguez Gutierrez, G. , J. Kim , A. H. Lee , et al. 2018. “Gene Signature of the Human Pancreatic Epsilon Cell.” Endocrinology 159: 4023–4032. 30380031 10.1210/en.2018-00833 PMC6963699

[acer70392-bib-0013] Engelstoft, M. S. , W. M. Park , I. Sakata , et al. 2013. “Seven Transmembrane G Protein‐Coupled Receptor Repertoire of Gastric Ghrelin Cells.” Molecular Metabolism 2: 376–392. 24327954 10.1016/j.molmet.2013.08.006 PMC3854997

[acer70392-bib-0014] Farokhnia, M. , M. L. Faulkner , D. Piacentino , M. R. Lee , and L. Leggio . 2019. “Ghrelin: From a Gut Hormone to a Potential Therapeutic Target for Alcohol Use Disorder.” Physiology & Behavior 204: 49–57. 30738971 10.1016/j.physbeh.2019.02.008

[acer70392-bib-0015] Farokhnia, M. , E. N. Grodin , M. R. Lee , et al. 2018. “Exogenous Ghrelin Administration Increases Alcohol Self‐Administration and Modulates Brain Functional Activity in Heavy‐Drinking Alcohol‐Dependent Individuals.” Molecular Psychiatry 23: 2029–2038. 29133954 10.1038/mp.2017.226 PMC12258500

[acer70392-bib-0016] Farokhnia, M. , G. Murphy , S. J. Weinstein , et al. 2021. “A Population‐Based Investigation of the Association Between Alcohol Intake and Serum Total Ghrelin Concentrations Among Cigarette‐Smoking, Non‐Alcohol‐Dependent Male Individuals.” Drug and Alcohol Dependence 226: 108835. 34214881 10.1016/j.drugalcdep.2021.108835 PMC8355123

[acer70392-bib-0017] Farokhnia, M. , J. Portelli , M. R. Lee , et al. 2020. “Effects of Exogenous Ghrelin Administration and Ghrelin Receptor Blockade, in Combination With Alcohol, on Peripheral Inflammatory Markers in Heavy‐Drinking Individuals: Results From Two Human Laboratory Studies.” Brain Research 1740: 146851. 32339499 10.1016/j.brainres.2020.146851 PMC8715722

[acer70392-bib-0018] Gersing, S. , T. Hansen , K. LINDORFF‐Larsen , and R. HARTMANN‐Petersen . 2025. “Glucokinase: From Allosteric Glucose Sensing to Disease Variants.” Trends in Biochemical Sciences 50: 255–266. 39753435 10.1016/j.tibs.2024.12.007

[acer70392-bib-0019] Godlewski, G. , R. Cinar , N. J. Coffey , et al. 2019. “Targeting Peripheral CB1 Receptors Reduces Ethanol Intake via a Gut‐Brain Axis.” Cell Metabolism 29: 1320–1333.e8. 31105045 10.1016/j.cmet.2019.04.012 PMC6551287

[acer70392-bib-0020] Goodyear, S. J. , M. Mottershead , E. Z. Sung , et al. 2010. “Dysregulation of Plasma Ghrelin in Alcoholic Cirrhosis.” Clinical Endocrinology 73: 323–329. 20184601 10.1111/j.1365-2265.2010.03793.x

[acer70392-bib-0021] Haque, L. Y. , and L. Leggio . 2024. “Integrated and Collaborative Care Across the Spectrum of Alcohol‐Associated Liver Disease and Alcohol Use Disorder.” Hepatology 80: 1408–1423. 38935926 10.1097/HEP.0000000000000996 PMC11841743

[acer70392-bib-0022] Jerlhag, E. , E. Egecioglu , S. Landgren , et al. 2009. “Requirement of Central Ghrelin Signaling for Alcohol Reward.” Proceedings of the National Academy of Sciences of the United States of America 106: 11318–11323. 19564604 10.1073/pnas.0812809106 PMC2703665

[acer70392-bib-0023] Kang, L. , B. M. Sebastian , M. T. Pritchard , B. T. Pratt , S. F. Previs , and L. E. Nagy . 2007. “Chronic Ethanol‐Induced Insulin Resistance Is Associated With Macrophage Infiltration Into Adipose Tissue and Altered Expression of Adipocytokines.” Alcoholism, Clinical and Experimental Research 31: 1581–1588. 17624994 10.1111/j.1530-0277.2007.00452.x

[acer70392-bib-0024] Kaur, S. , and A. E. Ryabinin . 2010. “Ghrelin Receptor Antagonism Decreases Alcohol Consumption and Activation of Perioculomotor Urocortin‐Containing Neurons.” Alcoholism, Clinical and Experimental Research 34: 1525–1534. 20586761 10.1111/j.1530-0277.2010.01237.x PMC2929279

[acer70392-bib-0025] Kharbanda, K. K. , M. Farokhnia , S. L. Deschaine , et al. 2022. “Role of the Ghrelin System in Alcohol Use Disorder and Alcohol‐Associated Liver Disease: A Narrative Review.” Alcoholism, Clinical and Experimental Research 46: 2149–2159. 36316764 10.1111/acer.14967 PMC9772086

[acer70392-bib-0026] Kim, D. J. , S. J. Yoon , B. Choi , et al. 2005. “Increased Fasting Plasma Ghrelin Levels During Alcohol Abstinence.” Alcohol and Alcoholism 40: 76–79. 15520048 10.1093/alcalc/agh108

[acer70392-bib-0027] Kojima, M. , H. Hosoda , Y. Date , M. Nakazato , H. Matsuo , and K. Kangawa . 1999. “Ghrelin Is a Growth‐Hormone‐Releasing Acylated Peptide From Stomach.” Nature 402: 656–660. 10604470 10.1038/45230

[acer70392-bib-0028] Koopmann, A. , C. VON DER Goltz , M. Grosshans , et al. 2012. “The Association of the Appetitive Peptide Acetylated Ghrelin With Alcohol Craving in Early Abstinent Alcohol Dependent Individuals.” Psychoneuroendocrinology 37: 980–986. 22172639 10.1016/j.psyneuen.2011.11.005

[acer70392-bib-0029] Kraus, T. , A. Schanze , M. Groschl , et al. 2005. “Ghrelin Levels Are Increased in Alcoholism.” Alcoholism, Clinical and Experimental Research 29: 2154–2157. 16385185 10.1097/01.alc.0000191753.82554.7e

[acer70392-bib-0030] Lee, M. R. , M. Farokhnia , E. Cobbina , et al. 2020. “Endocrine Effects of the Novel Ghrelin Receptor Inverse Agonist PF‐5190457: Results From a Placebo‐Controlled Human Laboratory Alcohol Co‐Administration Study in Heavy Drinkers.” Neuropharmacology 170: 107788. 31557492 10.1016/j.neuropharm.2019.107788 PMC7085971

[acer70392-bib-0031] Lee, M. R. , J. D. Tapocik , M. Ghareeb , et al. 2020. “The Novel Ghrelin Receptor Inverse Agonist PF‐5190457 Administered With Alcohol: Preclinical Safety Experiments and a Phase 1b Human Laboratory Study.” Molecular Psychiatry 25: 461–475. 29728704 10.1038/s41380-018-0064-y PMC6215751

[acer70392-bib-0032] Leggio, L. , A. Ferrulli , S. Cardone , et al. 2012. “Ghrelin System in Alcohol‐Dependent Subjects: Role of Plasma Ghrelin Levels in Alcohol Drinking and Craving.” Addiction Biology 17: 452–464. 21392177 10.1111/j.1369-1600.2010.00308.x PMC4974482

[acer70392-bib-0033] Leggio, L. , W. H. Zywiak , S. R. Fricchione , et al. 2014. “Intravenous Ghrelin Administration Increases Alcohol Craving in Alcohol‐Dependent Heavy Drinkers: A Preliminary Investigation.” Biological Psychiatry 76: 734–741. 24775991 10.1016/j.biopsych.2014.03.019 PMC4176606

[acer70392-bib-0034] Leko, A. H. , and L. Leggio . 2024. “Barriers to Alcohol Use Disorder Treatment in Patients With Alcohol‐Associated Liver Disease.” Clinics in Liver Disease 28: 779–791. 39362721 10.1016/j.cld.2024.06.012 PMC11458136

[acer70392-bib-0035] Li, Z. , G. Xu , Y. Qin , et al. 2014. “Ghrelin Promotes Hepatic Lipogenesis by Activation of mTOR‐PPARgamma Signaling Pathway.” Proceedings of the National Academy of Sciences of the United States of America 111: 13163–13168. 25157160 10.1073/pnas.1411571111 PMC4246976

[acer70392-bib-0036] Lu, X. , X. Zhao , J. Feng , et al. 2012. “Postprandial Inhibition of Gastric Ghrelin Secretion by Long‐Chain Fatty Acid Through GPR120 in Isolated Gastric Ghrelin Cells and Mice.” American Journal of Physiology. Gastrointestinal and Liver Physiology 303: G367–G376. 22678998 10.1152/ajpgi.00541.2011 PMC3774249

[acer70392-bib-0037] Mahalingam, S. , R. Bellamkonda , K. K. Kharbanda , et al. 2024. “Role of Ghrelin Hormone in the Development of Alcohol‐Associated Liver Disease.” Biomedicine & Pharmacotherapy 174: 116595. 38640709 10.1016/j.biopha.2024.116595 PMC11161137

[acer70392-bib-0038] Mahalingam, S. , R. Bellamkonda , K. K. Kharbanda , et al. 2026. “Characterizing Ghrelin's Role in Dysregulating GLP‐1‐Mediated Function and Promoting the Development of Alcohol‐Associated Liver Disease.” Biomedicine & Pharmacotherapy 195: 118924. 41518733 10.1016/j.biopha.2025.118924 PMC12906898

[acer70392-bib-0039] Mani, B. K. , and J. M. Zigman . 2017. “Ghrelin as a Survival Hormone.” Trends in Endocrinology and Metabolism 28: 843–854. 29097101 10.1016/j.tem.2017.10.001 PMC5777178

[acer70392-bib-0040] Mcvicker, B. L. , K. Rasineni , D. J. Tuma , M. A. Mcniven , and C. A. Casey . 2012. “Lipid Droplet Accumulation and Impaired Fat Efflux in Polarized Hepatic Cells: Consequences of Ethanol Metabolism.” International Journal of Hepatology 2012: 978136. 22506128 10.1155/2012/978136 PMC3312290

[acer70392-bib-0041] Muller, T. D. , R. Nogueiras , M. L. Andermann , et al. 2015. “Ghrelin.” Molecular Metabolism 4: 437–460. 26042199 10.1016/j.molmet.2015.03.005 PMC4443295

[acer70392-bib-0042] Naughton, S. S. , E. D. Hanson , M. L. Mathai , and A. J. Mcainch . 2018. “The Acute Effect of Oleic‐ Or Linoleic Acid‐Containing Meals on Appetite and Metabolic Markers; A Pilot Study in Overweight or Obese Individuals.” Nutrients 10: 1376. 30261617 10.3390/nu10101376 PMC6213143

[acer70392-bib-0043] Postic, C. , M. Shiota , and M. A. Magnuson . 2001. “Cell‐Specific Roles of Glucokinase in Glucose Homeostasis.” Recent Progress in Hormone Research 56: 195–217. 11237213 10.1210/rp.56.1.195

[acer70392-bib-0044] Ralevski, E. , T. L. Horvath , M. Shanabrough , R. Hayden , J. Newcomb , and I. Petrakis . 2017. “Ghrelin Is Supressed by Intravenous Alcohol and Is Related to Stimulant and Sedative Effects of Alcohol.” Alcohol and Alcoholism 52: 431–438. 28481974 10.1093/alcalc/agx022

[acer70392-bib-0045] Ramkissoon, R. , and V. H. Shah . 2022. “Alcohol Use Disorder and Alcohol‐Associated Liver Disease.” Alcohol Research: Current Reviews 42: 13. 36420303 10.35946/arcr.v42.1.13 PMC9662170

[acer70392-bib-0046] Rasineni, K. , J. L. Kubik , C. A. Casey , and K. K. Kharbanda . 2019. “Inhibition of Ghrelin Activity by Receptor Antagonist [d‐Lys‐3] GHRP‐6 Attenuates Alcohol‐Induced Hepatic Steatosis by Regulating Hepatic Lipid Metabolism.” Biomolecules 9: 517. 31546643 10.3390/biom9100517 PMC6843513

[acer70392-bib-0047] Rasineni, K. , P. G. Thomes , J. L. Kubik , E. N. Harris , K. K. Kharbanda , and C. A. Casey . 2019. “Chronic Alcohol Exposure Alters Circulating Insulin and Ghrelin Levels: Role of Ghrelin in Hepatic Steatosis.” American Journal of Physiology. Gastrointestinal and Liver Physiology 316: G453–G461. 30702902 10.1152/ajpgi.00334.2018 PMC6483023

[acer70392-bib-0048] Sakata, I. , W. M. Park , A. K. Walker , et al. 2012. “Glucose‐Mediated Control of Ghrelin Release From Primary Cultures of Gastric Mucosal Cells.” American Journal of Physiology, Endocrinology and Metabolism 302: E1300–E1310. 22414807 10.1152/ajpendo.00041.2012 PMC3361986

[acer70392-bib-0049] Sakata, N. , G. Yoshimatsu , and S. Kodama . 2019. “Development and Characteristics of Pancreatic Epsilon Cells.” International Journal of Molecular Sciences 20: 1867. 31014006 10.3390/ijms20081867 PMC6514973

[acer70392-bib-0050] Shankar, K. , S. Takemi , D. Gupta , et al. 2021. “Ghrelin Cell‐Expressed Insulin Receptors Mediate Meal‐ and Obesity‐Induced Declines in Plasma Ghrelin.” JCI Insight 6: e146983. 34473648 10.1172/jci.insight.146983 PMC8492315

[acer70392-bib-0051] Steensels, S. , L. Vancleef , and I. Depoortere . 2016. “The Sweetener‐Sensing Mechanisms of the Ghrelin Cell.” Nutrients 8: 795. 27941594 10.3390/nu8120795 PMC5188450

[acer70392-bib-0052] Stevenson, J. R. , J. M. Buirkle , L. E. Buckley , K. A. Young , K. M. Albertini , and A. E. Bohidar . 2015. “GHS‐R1A Antagonism Reduces Alcohol but Not Sucrose Preference in Prairie Voles.” Physiology & Behavior 147: 23–29. 25843741 10.1016/j.physbeh.2015.04.001

[acer70392-bib-0053] Stevenson, J. R. , L. M. Francomacaro , A. E. Bohidar , et al. 2016. “Ghrelin Receptor (GHS‐R1A) Antagonism Alters Preference for Ethanol and Sucrose in a Concentration‐Dependent Manner in Prairie Voles.” Physiology & Behavior 155: 231–236. 26723269 10.1016/j.physbeh.2015.12.017

[acer70392-bib-0054] Vannier, A. G. L. , J. E. S. Shay , V. Fomin , et al. 2022. “Incidence and Progression of Alcohol‐Associated Liver Disease After Medical Therapy for Alcohol Use Disorder.” JAMA Network Open 5: e2213014. 35594048 10.1001/jamanetworkopen.2022.13014 PMC9123494

[acer70392-bib-0055] Wittekind, D. A. , J. Kratzsch , R. Mergl , et al. 2018. “Alcohol Consumption Is Positively Associated With Fasting Serum Ghrelin in Non‐Dependent Adults: Results From the Population‐Based LIFE‐Adult‐Study.” Psychoneuroendocrinology 97: 143–148. 30029157 10.1016/j.psyneuen.2018.07.021

[acer70392-bib-0056] World Health Organization . 2018. Global status report on alcohol and health 2018. World Health Organization Licence: CC BY‐NC‐SA 3.0 IGO.

[acer70392-bib-0057] Yan, M. , S. Man , B. Sun , et al. 2023. “Gut Liver Brain Axis in Diseases: The Implications for Therapeutic Interventions.” Signal Transduction and Targeted Therapy 8: 443. 38057297 10.1038/s41392-023-01673-4 PMC10700720

[acer70392-bib-0058] Zhao, T. J. , I. Sakata , R. L. Li , et al. 2010. “Ghrelin Secretion Stimulated by beta1‐Adrenergic Receptors in Cultured Ghrelinoma Cells and in Fasted Mice.” Proceedings of the National Academy of Sciences of the United States of America 107: 15868–15873. 20713709 10.1073/pnas.1011116107 PMC2936616

[acer70392-bib-0059] Zhong, W. , Y. Zhao , Y. Tang , et al. 2012. “Chronic Alcohol Exposure Stimulates Adipose Tissue Lipolysis in Mice: Role of Reverse Triglyceride Transport in the Pathogenesis of Alcoholic Steatosis.” American Journal of Pathology 180: 998–1007. 22234172 10.1016/j.ajpath.2011.11.017 PMC3349880

[acer70392-bib-0060] Zhu, X. , Y. Cao , K. Voogd , and D. F. Steiner . 2006. “On the Processing of Proghrelin to Ghrelin.” Journal of Biological Chemistry 281: 38867–38870. 17050541 10.1074/jbc.M607955200

